# Autophagy in Cancer Therapy

**DOI:** 10.3389/fonc.2017.00128

**Published:** 2017-06-15

**Authors:** Simone Fulda

**Affiliations:** ^1^Institute for Experimental Cancer Research in Pediatrics, Goethe-University Frankfurt, Frankfurt, Germany; ^2^German Cancer Consortium (DKTK), Partner Site Frankfurt, Frankfurt, Germany; ^3^German Cancer Research Center (DKFZ), Heidelberg, Germany

**Keywords:** autophagy, cell death, cancer, autophagic cell death, cancer therapy

## Abstract

Autophagy represents a catabolic program involved in the degradation of cellular components *via* lysosomes. It serves to mitigate cellular stress and to provide metabolic precursors especially upon starvation. Thereby, autophagy can support the survival of cancer cells. In addition, there is now convincing evidence showing that under certain conditions autophagy can also foster cell death. This dual function of autophagy is also relevant upon anticancer treatment, as many chemotherapeutic agents engage autophagy. A better understanding of the molecular mechanisms that are critical for mediating autophagic cell death in cancer cells will be instrumental to selectively interfere with this cellular program in order to increase the cancer cell’s response to cytotoxic drugs. This review illustrates how anticancer drug-induced autophagy is involved in mediating cell death.

## Introduction

Autophagy is a basic cellular process that serves as a quality control checkpoint during physiological and pathophysiological conditions to cope with cellular stress. Autophagy regulates the turnover of damaged cellular elements that are degraded, for example, *via* lysosomal enzymes after engulfment into autophagosomes (1, 2). There are different forms of autophagy including macroautophagy (hereafter referred to as autophagy), microautophagy, and chaperone-mediated autophagy (3). A panel of genes involved in the regulation of autophagy has been identified over the last decades illustrating that autophagy represents a genetically controlled process (4). As far as cancer is concerned, autophagy may function as both a tumor suppressor and tumor promoter (5). One explanation is the dual function of autophagy being either cytoprotective or cytotoxic in a context-dependent fashion. By definition, autophagic cell death (ACD) refers to a mode of cell death that is inhibited *via* specific blockage of the autophagic pathway (6).

Anticancer treatments can engage autophagy in cancer cells on the one side as part of a cytoprotective answer in response to a toxic insult with the aim to mitigate cellular stress (7). On the other side, anticancer therapy can stimulate autophagy pathways that mediate ACD (8). In the following, prototypic examples of ACD upon anticancer treatments will be discussed.

## Anticancer Drug-Induced ACD

### Chemotherapeutic Drugs

Several chemotherapeutic drugs have been reported to engage autophagy (9–12). While chemotherapy-mediated autophagy has mostly been linked to a cytoprotective response that allows cancer cells to cope with the cellular stress imposed upon anticancer drug treatment, there are also cases of ACD. For example, the DNA-alkylating agent temozolomide (TMZ) has been implicated to elicit ACD. TMZ belongs to the class of DNA-alkylating drugs that triggers the formation of *O*-6-methylguanine in DNA, which causes DNA damage during the following cycle of DNA replication by mispairing with thymine. TMZ has been shown to trigger the recruitment of LC3 to autophagosomal membranes (9). Pharmacological inhibition of autophagy by 3-methyladenine (3-MA) resulted in reduced formation of autophagosomes and attenuated TMZ-mediated cytotoxicity (9). In addition, genetic inhibition of autophagy by RNAi-mediated gene silencing of ATG5 and Beclin 1 (BECN1) impaired cell death upon treatment with TMZ alone or in combination with (−)-gossypol (13), underscoring that autophagy may contribute to TMZ-imposed cytotoxicity. However, a cytoprotective function of autophagy in the course of chemotherapy has also been proposed. This conclusion is based on data showing that a TMZ-induced and autophagy-dependent increase of ATP counteracts cell death of malignant glioma upon exposure to TMZ (14). Consistently, knockdown of core elements of autophagy signaling such as BECN1 enhanced the sensitivity of malignant glioma cells to TMZ-imposed reduction of colony formation after TMZ treatment (14). However, the question as to whether or not TMZ induces ACD remains a controversially discussed issue, since TMZ has also been reported to trigger apoptosis (10). Thus, further studies are required to determine the functional relevance of autophagy in the course of TMZ-induced antitumor activity in malignant glioma cells.

### BH3 Mimetics

BH3 mimetics that antagonize antiapoptotic BCL-2 family proteins have been reported to engage ACD by disrupting a complex of BECN1/ATG6 together with BCL-2 or BCL-x_L_ (15–17). One example is gossypol, a natural compound derived from cotton seeds that functions as a pan-BCL-2 inhibitor by neutralizing BCL-2, BCL-x_L_, MCL-1, and BCL-w. (−)-Gossypol (also known as AT-101) proved to be the more potent enantiomer of gossypol to suppress tumor growth as compared to (+)-gossypol. In apoptosis-deficient cancer cells, gossypol has been reported to induce ACD (13, 18), while it triggered apoptotic cell death in cells that can undergo apoptosis (19–22). In glioblastoma cells, (−)-gossypol reportedly triggered ACD alone and in combination with the alkylating agent TMZ, which was accompanied by translocation of LC3 to autophagosomes, and lysosomal activity (13). ACD was supported by rescue experiments demonstrating that knockdown of BECN1 or ATG5 significantly reduced (−)-gossypol-induced cell death alone and combined treatment with TMZ (13). Besides glioblastoma, (−)-gossypol was shown to trigger ACD in apoptosis-resistant prostate cancer and breast carcinoma cells, as silencing of ATG5 and BECN1 significantly rescued (−)-gossypol-mediated cell death.

### Obatoclax

Furthermore, obatoclax has been implicated in triggering ACD and the conclusion that it is in fact ACD contributing to obatoclax-induced cell death was drawn on findings showing that genetic silencing of essential autophagy genes such as BECN1, ATG5, or ATG7 inhibits obatoclax-mediated cell death. For example, obatoclax has been shown to exert antileukemic activity in pediatric acute lymphoblastic leukemia including glucocorticoid-resistant cases by engaging autophagy and cell death (23). Parallel silencing of autophagy-related genes such as BECN1 or ATG7 provided protection against obatoclax, underscoring that autophagy is indeed necessary for the observed antileukemic activity (23). Moreover, obatoclax has been reported to stimulate the assembly of the necrosome on autophagosomes, thereby linking autophagy to necroptotic cell death (24). Silencing of ATG5 or ATG7 rescued cells from obatoclax-mediated cell death, highlighting their requirement for cell death upon treatment with obatoclax (24). Also, obatoclax has been described to trigger the conversion of LC3 and cell death in a BECN-dependent manner in B-cell lymphoma (25). Besides autophagy, obatoclax has also been shown to engage apoptosis (26, 27).

### Cannabinoids

Tetrahydrocannabinol (THC), which is considered as the main active component of cannabinoids, has been shown to act as a stimulus for ACD, for example, in hepatocellular carcinoma (HCC) and glioblastoma (28, 29). Knockdown of ULK1, ATG5, or Ambra-1 conferred protection of glioblastoma cells from THC-induced cell death (28). Similarly, ATG5 knockout fibroblasts were shown to be refractory to THC-stimulated cytotoxicity (28). In addition, ATG5 deficiency rescued THC-triggered antitumor activity in a tumor xenograft model *in vivo* (28). These studies confirmed the contribution of autophagy to THC-mediated antitumor activity both *in vitro* and *in vivo*.

Molecular studies revealed that THC causes ER stress *via* accumulation of ceramide and phosphorylation of eukaryotic translation initiation factor 2 alpha, resulting in upregulation of CHOP and tribbles homolog 3 (TRB3), two ER stress-related proteins (28). TRB3 then engages autophagy by blocking AKT/mTOR signaling (28). In sharp contrast to THC-triggered ACD in various types of cancer cells, THC did not possess a similar cytotoxicity against normal non-malignant cells (28). This indicates that THC preferentially targets cancer rather than normal cells and thus may offer a therapeutic window that could be exploited for cancer therapy.

JWH-015 is a cannabinoid receptor 2-selective agonist that has been shown to engage ACD in HCC that involved AMPK activation and inhibition of AKT/mTOR signaling (29). Of note, ATG5 silencing or 3-MA protected from JWH-015-induced reduction of HCC growth (29).

## Histone Deacetylase Inhibitors (HDACIs)

Histone deacetylase inhibitors represent another class of anticancer agents that have been reported to engage autophagy associated with the induction of cell death in chondrosarcoma cell lines. Suberoylanilide hydroxamic acid (SAHA) has been shown to stimulate autophagy-associated cell death accompanied by ultrastructural changes in autophagosome formation and increased lipidation of LC3 (30). Pharmacological inhibition of autophagy using 3-MA significantly protected from SAHA-mediated loss of cell viability (30). However, no genetic evidence has been provided in this study to confirm that autophagy is indeed required for the induction of cell death. Thus, it remains to be confirmed that SAHA in fact triggers ACD in chondrosarcoma cells.

In HeLa cervical carcinoma cells, SAHA has been reported to induce characteristic autophagic features including morphological changes and LC3-II conversion (31). Genetic silencing of BECN1 and ATG7 inhibited SAHA-stimulated autophagy (31). However, the question as to whether or not autophagy genes are also required for SAHA-induced cell death has not yet been answered (31).

In HCC, HDACIs including SAHA and OSU-HDAC-42 have been described to trigger ACD based on both genetic- and pharmacological blocking experiments underscoring that SAHA- or OSU-HDAC-42-stimulated autophagy is required for the induction of cell death, as either silencing of ATG5 or 3-MA protected cells from the cytotoxicity of SAHA (32). Also, autophagosome formation, LC3 lipidation, and downregulation of p62 have been observed upon treatment with SAHA (32). SAHA and OSU-HDAC-42 might stimulate autophagy by blocking the mTOR pathway, as they suppress AKT/mTOR activity (32).

## New Combinations

The tricyclic antidepressant (TCA) imipramine (IM) and the anticoagulant ticlopidine (TIC), two drugs approved by the US Food and Drug Administration, have been shown to synergistically trigger autophagy and cell death in glioblastoma cells (33). In addition, this combination proved to be effective to suppress glioblastoma growth in a murine *in vivo* model. ACD was emphasized by genetic knockdown of ATG7 that significantly rescued combination treatment-induced cell death. The authors went on to show that IM and TIC increase the autophagic flux by upregulating 3′-5′-cyclic adenosine monophosphate (cAMP) levels *via* distinct mechanisms. While IM treatment activates adenylate cyclase and induces cAMP-mediated autophagy, the addition of the P2Y_12_ inhibitor TIC short circuits the ADP/ATP-induced feedback inhibition of adenylate cyclase (33). Together, this increases cAMP levels and elicits hyperactivated autophagy and subsequent cell death. It is interesting to note that the clinical use of TCAs has previously been associated with a decreased incidence of glioblastoma.

## Conclusion

There is ample evidence showing that some cytotoxic drugs used for the treatment of cancer can engage ACD. Since this property can in principle be exploited for cancer therapy, it is critical to understand the pathways regulating these events. However, as autophagy can control both cell death and survival programs, induction of autophagy in cancer cells represents a double-edged sword. It will therefore be critical to develop novel approaches that will allow selective engagement of the pro-death branch of autophagy.

## Author Contributions

SF has solely contributed to the conception and design of the work, has drafted and revised the work, and has given final approval of the version to be published.

## Conflict of Interest Statement

The author declares that the research was conducted in the absence of any commercial or financial relationships that could be construed as a potential conflict of interest.
